# Immunomodulation of the Prostate Tumor Microenvironment Following Inorganic Arsenic Exposure

**DOI:** 10.1002/jat.70108

**Published:** 2026-02-23

**Authors:** Joseph J. Shearer, Cosette Rivera‐Cruz, Alexandre M. B. Cavalca, Carlos Eduardo Fonseca‐Alves, Marxa L. Figueiredo

**Affiliations:** ^1^ Department of Basic Medical Sciences, College of Veterinary Medicine Purdue University West Lafayette Indiana USA; ^2^ Department of Veterinary Surgery and Animal Reproduction São Paulo State University (UNESP) Botucatu SP Brazil; ^3^ Department of Veterinary Surgery, School of Veterinary Medicine and Animal Science University of São Paulo‐USP São Paulo Brazil; ^4^ Institute of Health Sciences Paulista University (UNIP) Bauru SP Brazil

## Abstract

The tumor microenvironment (TME) influences prostate cancer (PCa) progression through stromal and immune interactions. Adipose‐derived mesenchymal stromal cells (ASCs) modulate immune tone, while inorganic arsenic (iAs), a widespread toxicant, is linked to immune suppression and carcinogenesis. Their combined impact on PCa immunity has remained unclear. Using a Ras‐driven murine PCa model (TC2Ras, which mimics aggressive, immune‐interactive PCa through constitutive Ras signaling), we assessed ASC and chronic iAs exposure effects on tumor growth, immune infiltration, and transcriptomic remodeling via flow cytometry, RNA‐seq, and qPCR. ASC‐conditioned media increased TC2Ras viability by up to 82%, an effect reversed by iAs (300–1000 ppb). In vivo, ASC co‐implantation significantly elevated tumor weight in ASC + iAs tumors. ASC promoted approximately twofold macrophage and CD4^+^ T‐cell infiltration, while iAs suppressed macrophages and MDSCs. We performed RNA‐seq and qPCR, confirming that a sustained IFNγ‐IRF1 activation (approximately eightfold) in ASC tumors occurred alongside an iAs‐driven downregulation of adaptive immunity, as well as an upregulation of immune checkpoint genes (Pdcd1, Lag3). These findings demonstrate that ASC–iAs crosstalk remodels the TME toward immune tolerance and chronic IFNγ signaling, potentially facilitating tumor progression and revealing novel mechanisms by which environmental toxicants may influence cancer immunity through stromal cell interactions.

## Abstract 2

The tumor microenvironment shapes how prostate cancer (PCa) progresses through stromal‐immune interactions. We investigated how adipose‐derived mesenchymal/stromal cells (ASC) and chronic inorganic arsenic (iAs) exposure jointly influence PCa by using a Ras‐driven murine model. ASC‐conditioned media enhanced TC2Ras viability, while iAs reversed this effect. In vivo, ASC + iAs tumors showed increased weight, altered immune infiltration, and transcriptomic remodeling, including a sustained IFNγ‐IRF1 axis and immune checkpoint upregulation. ASC–iAs crosstalk promotes immune tolerance, revealing mechanisms by which environmental toxicants modulate cancer immunity.

**Abbreviations**
ASC: Adipose‐derived stromal cell
Arg1: Arginase 1
CD: Cluster of differentiation
CM: Conditioned media
DEG: Differentially expressed gene
F4/80: Murine macrophage marker
FMO: Fluorescence minus one
FPKM: Fragments per kilobase of transcript per million mapped reads
GEO: Gene expression omnibus
IFNγ: Interferon gamma
IL‐1β: Interleukin 1 beta
iAs: Inorganic arsenic
iNOS: Inducible nitric oxide synthase
IRF1: Interferon regulatory factor 1
Ly6C: Monocyte marker
MCODE: Molecular complex detection
MDSC: Myeloid‐derived suppressor cell
M‐MDSC: Monocytic myeloid‐derived suppressor cell
MSC: Mesenchymal stromal cell
NK Cell: Natural killer cell
NKT: Natural killer T cell
PCa: Prostate cancer
qRT‐PCR: Quantitative reverse transcription polymerase chain reaction
RIN: RNA integrity number
RNA‐seq: RNA sequencing
TC2Ras: Ras‐driven prostate cancer cell line
Tfh: T follicular helper cell
TGFβ: transforming growth factor beta
Th1: T helper type 1 cell
TIMER2.0: Tumor immune estimation resource
TME: Tumor microenvironment
TPM: Transcripts per million
TRAMP: Transgenic adenocarcinoma of mouse prostate
TRRUST: Transcriptional regulatory relationships unraveled by sentence‐based text mining
Treg: regulatory T cell

## 1 Introduction

The tumor microenvironment (TME) can be considered a complex milieu of cell types including epithelial (normal or tumor cells), endothelial, stem, and immune cells, among others. For instance, in prostate cancer (PCa), the TME is characterized by a unique fibromuscular stroma and epithelial cells which are androgen‐responsive and able to interact dynamically with both the immune and the stromal components (Pederzoli et al. 2023). Each of these cell populations influences cell signaling within the TME through the release of cytokines and growth factors that, when uncontrolled, can facilitate the progression of cancer (Quail and Joyce 2013). These soluble factors influence the tumor directly and indirectly through the recruitment and maturation of immune cells. However, the TME, rather, can attenuate the function of recruited immune cells, creating an immunosuppressive environment that is conducive to tumor progression (Du et al. 2024; Giraldo et al. 2019; Goliwas et al. 2021). This has been shown in the clinic for PCa tissues, whose prostate stroma creates an immunosuppressive environment strongly associated with tumor progression (Ribeiro, Monteiro, Catalán, et al. 2012). Given that PCa is one of the most prevalent malignancies in men and that it often progresses silently until reaching the advanced stages, understanding the immunosuppressive mechanisms within its TME is critical for developing effective therapies.

Adipose‐derived mesenchymal stromal cells (ASCs) are enriched in tumors of PCa patients (Ribeiro, Monteiro, Silvestre, et al. 2012), in part, to restore a homeostatic state within the TME by dampening inflammatory responses (Hocking 2015). However, our group (Shearer et al. 2016) and others (Prantl et al. 2010) have shown that ASCs can influence cancer cell growth, and this is partly through the release of soluble factors. It is known that the paracrine signaling from mesenchymal stromal cells (MSCs) contributes to the recruitment of immune cells to the tumor (Sun et al. 2014) and can alter proliferation rates and maturation of several immune cell populations (Cuiffo and Karnoub 2012). In the context of PCa, ASCs may contribute to tumor progression not only via immunomodulation but also by promoting angiogenesis and to some extent also through epithelial‐mesenchymal transition (Liang et al. 2021; Su et al. 2021).

Genetically engineered mouse models, such as TC2Ras and TRAMP, have provided valuable platforms for studying PCa progression (Figueiredo et al. 2024; Kido et al. 2019). For example, the TRAMP model uses the probasin promoter to drive SV40 T antigen expression in the prostate epithelium, and this model can recapitulate several features of human PCa, including neuroendocrine differentiation and metastatic spread. Meanwhile, the TC2Ras model developed by our group incorporated oncogenic Ras signaling and has been shown to enable recruitment of a variety of tumor‐promoting cell components (Figueiredo et al. 2021). This model thus enables the study of epithelial‐stromal interactions and immune modulation within the TME (Kumar et al. 2025). TRAMP and TC2Ras are among models that can be used for dissecting the influence of environmental exposures such as inorganic arsenic (iAs) on tumor biology and immune cell dynamics. By employing these models, we can better understand how stromal and immune components respond to carcinogenic stimuli and how they may contribute to PCa progression.

Largely understudied areas of tumor biology include not only the understanding of the complex interplay between stromal cell populations and PCa cells within the TME but also the susceptibility of the TME to environmental exposures. iAs represents a major public health concern, with > 200 million people yearly exposed to unsafe levels in their drinking water alone (Bommarito et al. 2019; Frisbie and Mitchell 2022; Naujokas et al. 2013; Smeester et al. 2017). Arsenic and iAs compounds are classified as Group 1 carcinogens by the International Agency for Research on Cancer (IARC), reflecting sufficient evidence of carcinogenicity in humans (IARC Working Group on the Evaluation of Carcinogenic Risks to Humans 2012). And although iAs has been implicated in the development of several cancers, including PCa (Alvarado‐Morales et al. 2021; Escudero‐Lourdes et al. 2022; Xu et al. 2021), its potential role in modulating cell‐mediated signaling within the prostate TME has remained poorly understood. Therefore, in this study, we examined the hypothesis that exposure to iAs could alter the ASC‐mediated recruitment and/or maturation of immune cells within the TME, thus influencing prostate tumor growth.

## 2 Materials and Methods

### 2.1 Cell Culture and Conditioned Media (CM) Collection

Mouse ASC‐GFP primary cells, alongside NIH 3T3 (fibroblastic), 3T3 L1 (preadipocyte‐like), and differentiated 3T3 L1 (adipocyte‐like) lines representing a spectrum of adipocyte differentiation, were maintained and utilized as described below. ASC‐GFP cells were isolated from 8‐ to 12‐week‐old male C57BL/6‐Tg (UBC‐GFP) mice using established protocols (21). ASCs were cultured as described in “base media” (21,22), while NIH 3T3 and 3T3 L1 were cultured in DMEM with 10% FBS and 1X anti‐anti. Prior to iAs exposure, 3T3 L1 cells were differentiated into adipocyte‐like cells as described (Reed and Lane 1980). To model the periprostatic adipose stromal spectrum, each cell type was exposed to 0, 300, or 1000 ppb iAs in base media for 1 week, followed by washing and replacement with DMEM:F12 with 2% FBS for 24 h before collecting the CM. The iAs concentrations were selected (300 and 1000 ppb) based on (i) the rationale of the environmentally relevant chronic exposure levels reported in contaminated groundwater and (ii) to align with prior studies demonstrating stromal and immune modulation without systemic toxicity (9,30).

### 2.2 Cell Viability

The mouse PCa cell line TC2Ras was previously generated and characterized by our group (Figueiredo et al. 2024; Umbaugh et al. 2018; Zolochevska et al. 2013) and cultured as described (Figueiredo et al. 2024). TC2Ras were seeded at 3000 cells per well in 96‐well plates. After 24 h, baseline cell viability was determined using the Cell Counting Kit 8 (CCK‐8 assay) (Dojindo), with analyses as we have previously reported (Zolochevska and Figueiredo 2011). Media was replaced with CM collected from each of the adipogenic‐like lineages at a defined cell:cell ratio (from 0% to 10%); TC2Ras cells were incubated with CM for 48 h, with cell viability reassessed using CCK‐8.

### 2.3 Animal Exposure and Tumor Implantation

All animal procedures were performed under veterinary supervision per protocols approved by the Purdue University's Institutional Animal Care and Use Committee (PACUC). Mice initially received low‐arsenic chow (AIN‐76A purified diet [Research Diets Inc.]) and Milli‐Q water for 2 weeks to flush residual iAs. Mice were then temporarily anesthetized with isoflurane and subcutaneously injected with 125,000 TC2Ras ± 1% ASC‐GFP cells into the flanks. ASC proportion rationale was based on our prior studies (Shearer et al. 2017; Shearer et al. 2016) and aligns with MSC levels reported in human prostatectomy tissue (Brennen et al. 2016; Brennen et al. 2013). Postimplantation, mice were split to receive either 0 or 300 ppb of iAs in the drinking water for the duration of the study. Tumors were monitored twice weekly by caliper measurements of width and perpendicular length, with volume estimated by the following: Tumor volume (mm^3^) = 0.5 × (length [mm] × width [mm]^2^).

### 2.4 Tumor Isolation

At 21 and 26 days posttumor cell implantation, mice (*n* = 3–8 per group) were euthanized by CO₂. Tumors were excised, weighed, and halved for RNA and flow cytometry analyses, combining left and right flank samples per mouse. Tumor samples were kept in 1X PBS until processing. A dissociation solution (~1.3 mL) containing 2.5 mg/mL of DNase II, 1 mg/mL of collagenase IV, and 0.5 mg/mL of hyaluronidase V (in PBS, without Mg/Ca) was added to tumor minced sections made utilizing scalpels. Samples were incubated at 37°C in the presence of shaking (at ~90 rpm) for 90 min, then filtered utilizing a 70‐μm strainer. Cells were washed twice with PBS (with Ca^2+^/Mg^2+^) and stored on ice before proceeding to flow cytometry staining (Zolochevska et al. 2011).

### 2.5 Flow Cytometry

Tumor‐derived cells (200,000/sample) were stained to assess viability using the LIVE/DEAD Fixable dye (1 μL, 30 min on ice), followed by surface antibody panels (Table S1) for 30 min at 4°C. After this, cells fixed in 2% paraformaldehyde (PFA) were stored at 4°C for ≤ 4 days. Intracellular staining was performed after permeabilization (Permeabilization Wash Buffer, BioLegend) with antibodies (Table S1) for 30 min at room temperature (RT). After washing and filtering through a 35‐μm strainer, samples were analyzed on a BD Fortessa Cell Analyzer at Purdue's Flow Cytometry Facility. Compensation was calculated using UltraComp eBeads and gating employed Fluorescence Minus One (FMO) controls (Tung et al. 2004) to delineate gates accounting for spectral overlap from multiple fluorochromes. Data analyses were performed with FlowJo v10 (FlowJo LLC). T cell subsets were identified using CD4, CD8, and TCR γδ markers, following multiparametric flow cytometry panels described in mice (Buus et al. 2019). Macrophages were characterized by F4/80 and CD11b; CD38 and Egr2 were used to distinguish M1 and M2 phenotypes based on established markers in murine macrophages (Jablonski et al. 2015). MDSCs were defined by CD11b^+^Gr‐1^+^ and further subdivided into Ly6C^+^ (monocytic or M‐MDSCs) and Ly6G^+^ (granulocytic or G‐MDSCs) subsets, as detailed in standard mouse MDSC protocols (Höchst et al. 2015).

### 2.6 RNA Extraction

To preserve RNA integrity, tumor fractions were immersed in RNAlater Stabilization Solution (Invitrogen) and stored at 4°C for up to 7 days following manufacturer guidelines (Grotzer et al. 2000). Afterwards, tissues were transferred to −80°C for long‐term storage. Approximately 15–18 mg of tissue was used for RNA extraction. Samples were homogenized in 600 μL of RLT buffer with 10 μL of β‐mercaptoethanol using a PRO200 homogenizer (three 10‐ to 15‐s pulses at medium power). For in vitro cell pellets, 350 μL of RLT buffer was used and lysates were passed three times through a 27G syringe. All lysates were processed with the Qiagen RNeasy Mini Kit, with RNA eluted in 50 μL of DNase/RNase‐free water (Invitrogen).

### 2.7 RNA Sequencing and Bioinformatics Analyses

To gain an understanding of the molecular and immunological changes induced by ASC and/or iAs exposure in TC2Ras tumors, we performed RNA sequencing (RNA‐seq) on tumor samples collected 21 days postimplantation. This time point was selected because gene expression changes had stabilized as detected with qPCR pilot studies and were significantly different from controls (at Day 26, tumor overgrowth obscured group differences). RNA‐seq was performed in collaboration with LC Sciences (Houston, TX, USA), with prior assessment of RNA integrity using the Agilent 2100 Bioanalyzer; all samples had RIN scores > 7.0. Libraries were prepared with Illumina's TruSeq Stranded mRNA kit, which enriches poly(A) RNA via oligo (dT) beads, followed by fragmentation with divalent cations at elevated temperature. Library quality was validated on the Agilent Bioanalyzer. Sequencing was conducted on the Illumina NovaSeq 6000 with paired‐end reads. Raw reads were trimmed of adapters, low‐quality, and ambiguous bases using Cutadapt and custom Perl scripts, with quality control via FastQC. Clean reads were aligned to the *Mus musculus* genome using HISAT2, then assembled with StringTie, merged with gffcompare, and quantified with StringTie and Ballgown, reporting FPKM values. Differential expression analysis was performed with DESeq2 (Liu et al. 2021) and edgeR (Robinson et al. 2010), considering genes with adjusted *p*‐value < 0.05 and|fold change | ≥ 1.5 as significant.

### 2.8 Functional Enrichment and Pathway Analysis

To best interpret the biological significance of differentially expressed genes, we conducted enrichment analyses using Metascape (https://metascape.org/) (Zhou et al. 2019). Gene lists were uploaded with *M. musculus* selected as the reference species, and all available annotation categories, which include pathways, structural complexes, and functional sets, were included. Custom parameters were set to a minimum overlap of three genes and a *p*‐value < 0.05. Protein–protein interaction (PPI) enrichment was assessed using combined databases. Additionally, g:Profiler (https://biit.cs.ut.ee/gprofiler/) was used to explore regulatory networks, particularly transcription factor enrichment among upregulated genes, using the g:GOSt module and multiple testing correction.

### 2.9 Immune Cell Composition Estimation

To assess immune cell infiltration in tumors posttreatment, immune deconvolution was performed using TIMER2.0 (Li et al. 2020), which accepts TPM‐normalized RNA‐seq data as input. Developed for TME analysis, TIMER2.0 incorporates the immunedeconv R package, which integrates multiple deconvolution algorithms applicable to a variety of tissue types including tumors.

### 2.10 RT‐qPCR Analysis

Complementary DNA was synthesized from 0.5 μg total RNA using amfiRivert Platinum cDNA Synthesis Master Mix (GenDepot). Quantitative PCR used 1 μL of cDNA, 2× KAPA SYBR Fast Master Mix (Roche), and 1 to 2 μM gene‐specific primers, with GAPDH as the internal control. Amplification was performed on a ViiA7 and QuantStudio 3 systems (Thermo Fisher Scientific) under cycling: 95°C for 3 min; 40 cycles of 95°C for 3 s, 60°C for 30 s, and 72°C for 19 s. Data were analyzed using QuantStudio 3 software.

### 2.11 Statistical Analysis

Statistical calculations were performed using GraphPad Prism. Conditioned media (CM) experiments and TC2Ras viability were assessed for statistically significant relationships utilizing one‐way ANOVA analyses. For determining statistical significance for estimated tumor size, tumor mass, flow cytometry, and RT‐qPCR results, the exposure and commix groups were assessed using a two‐way ANOVA, with *p* < 0.05 considered significant. Results are shown as the mean ± SD with individual samples shown as dots with bars representing the max/min samples.

## 3 Results

### 3.1 In Vitro Modeling of the TME and the Effects of iAs and ASCs

**FIGURE 1:**
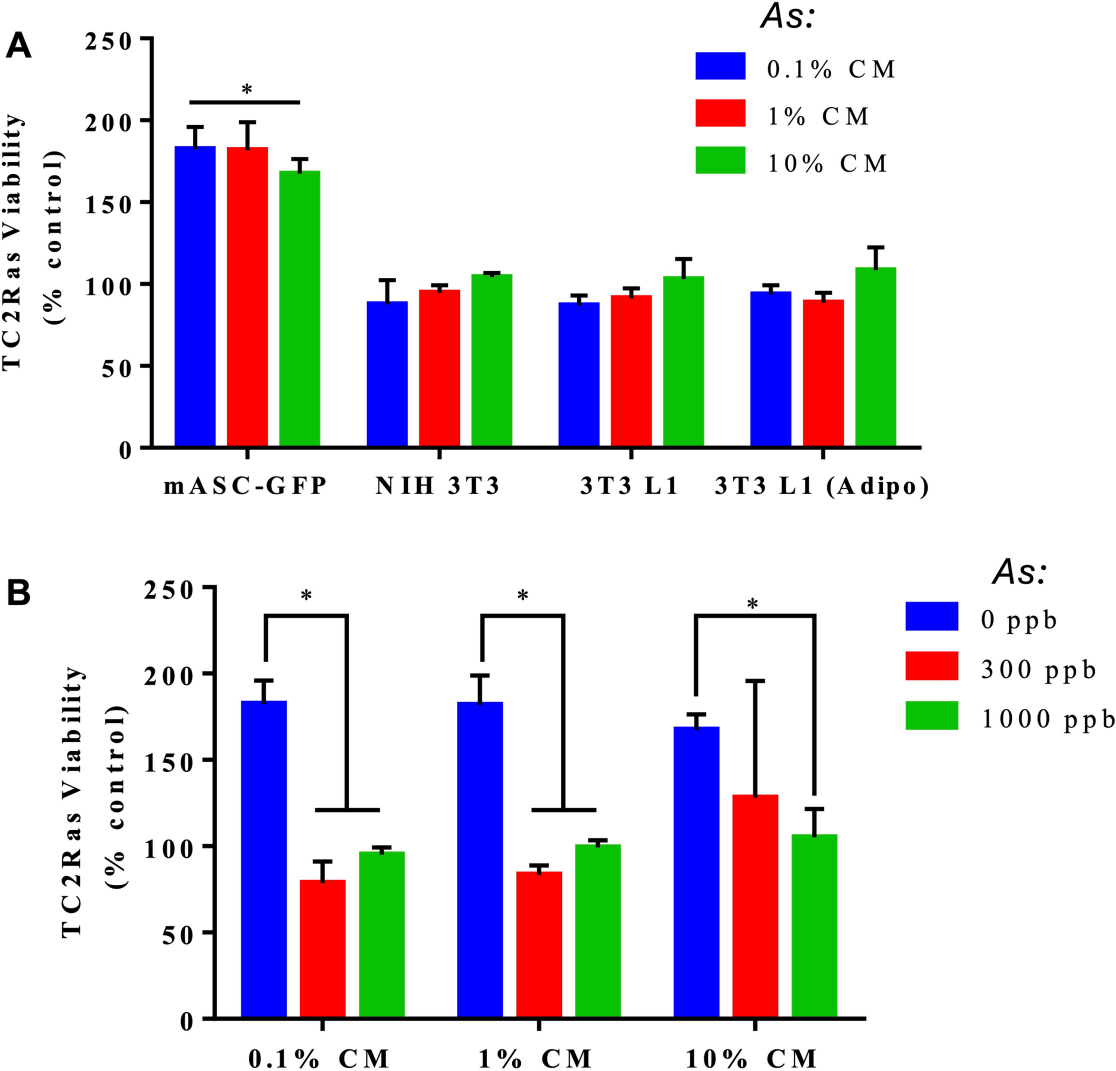
Effects of adipocyte‐like lineage CM and iAs exposure on TC2Ras cell viability. (A) TC2Ras viability after 48 h exposure to 0.1%–10% CM from ASC‐GFP (stem), NIH 3T3 (fibroblastic), 3T3 L1 (preadipocyte‐like), and differentiated 3T3 L1 (adipocyte‐like) cells. Data are shown as mean ± SD relative to 0% CM control; *p < 0.05 by one‐way ANOVA. (B) TC2Ras viability after 48 h exposure to 0.1%–10% CM from ASC‐GFP pretreated with 0, 300, or 1000 ppb iAs for 7 days. Data are shown as mean ± SD relative to 0 ppb of CM control; *p < 0.05 by one‐way ANOVA.

To examine how the differentiation status of stromal TME components affects cancer cell viability, we cultured TC2Ras PCa cells with conditioned media (CM) from various adipocyte‐like cells. Only CM from the stem/progenitor population (ASC‐GFP) was able to significantly increase TC2Ras viability by 48 h post‐exposure (Figure 1A), with 0.1%–10% CM inducing a 67%–82% viability increase versus control. Subsequently, we evaluated iAs impact on the ASC secretome effect. The ASC CM‐induced increase in TC2Ras viability was attenuated after 1 week of iAs exposure (Figure 1B). Exposure to 300 and 1000 ppb iAs restored viability toward baseline in 0.1% and 1% CM, whereas in 10% CM samples, only 1000 ppb iAs significantly reduced viability. These results suggest that the ASC secretome may enhance PCa cell viability, yet that this effect is dose‐dependently mitigated by iAs exposure. For determining the ASC proportions, 1% ASC‐GFP cells were chosen based on our prior studies (9,30) and the reported MSC frequencies in human tissue, as characterized from human prostatectomy samples (31,32), for physiological relevance.

### 3.2 The Interaction Between iAs and ASC on Tumor Burden

**FIGURE 2:**
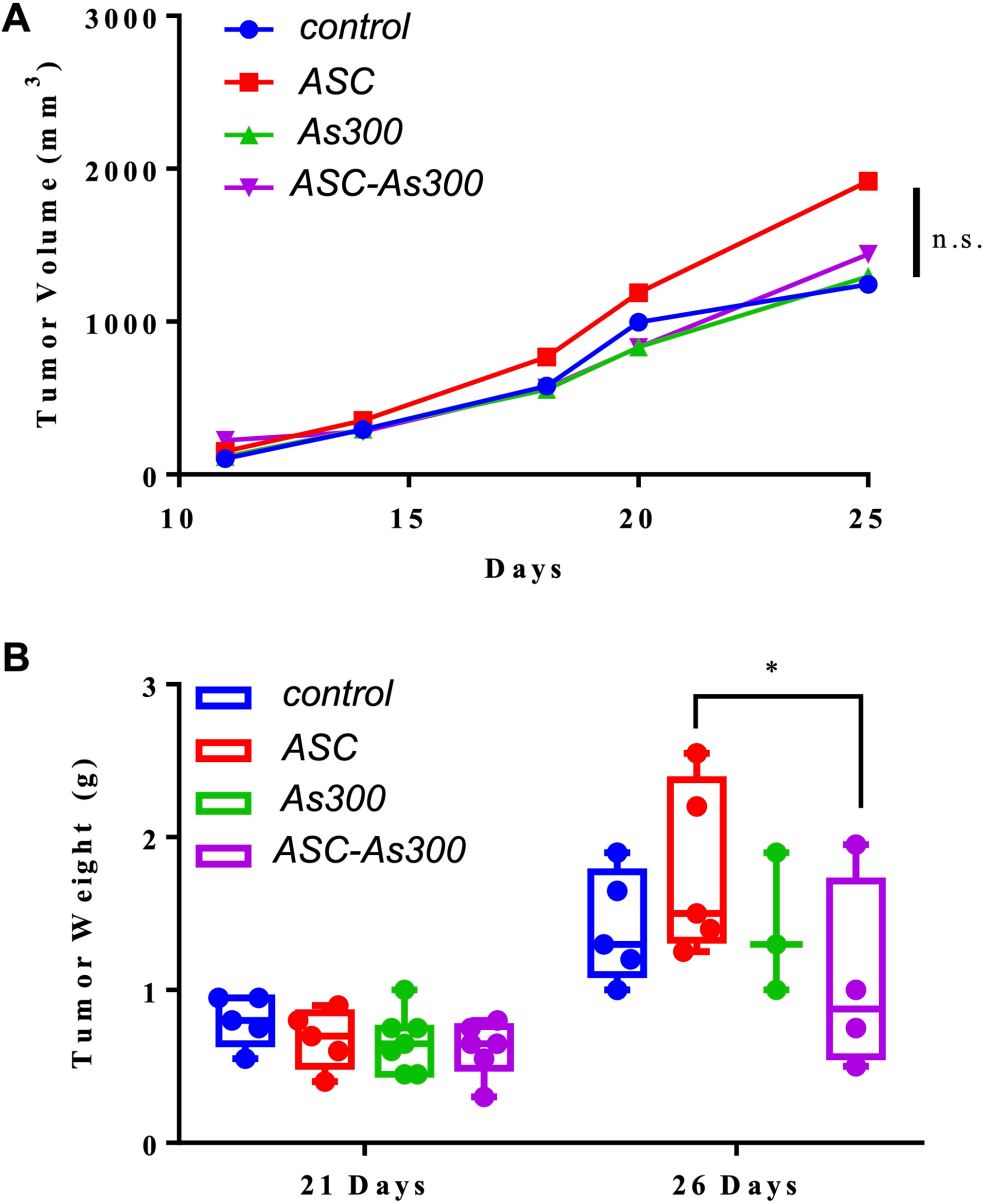
Subcutaneous prostate tumor characteristics comparing ASC commixing and arsenic exposure. (A) Data showsthe average estimated tumor size as measured by calipers over the course of the study. Error bars were ommitted for clarity and no signifcance (N.S.) was observed when comparing any of the four groups at any timepoints. (B) Box and whisker plot with individual samples shown as dots for average tumor data with the bars representing the max/min samples. Statistically significant differences (*, *p* < 0.05) as assessed by two‐way ANOVA.

To assess how iAs and ASC might interact to influence tumor growth, we implanted TC2Ras cells ± 1% ASC in mice (exposed to 0 or 300 ppb iAs, with continued exposure throughout the study). Although tumor volumes did not differ significantly across groups (Figure 2A), the 0 ppb iAs + 1% ASC group consistently showed larger tumors, particularly at the later time points (1188 and 1920 mm^3^ at Days 20 and 25 relative to 899 and 1330 mm^3^ in other groups). This pattern suggests that ASCs may promote tumor growth under baseline conditions, while iAs exposure, in contrast, dampens this effect. Tumor weights were measured at 21 and 26 days postimplantation to complement caliper‐based volume estimates (Figure 2B). At Day 21, average tumor weights were similar across TC2Ras ± 1% ASC groups with or without iAs (0.62–0.80 g), showing no significant differences, yet by Day 26, weights increased in all groups (1.05–1.78 g), with a significant difference (*p* = 0.02) observed between 0 ppb iAs + 1% ASC and 300 ppb iAs + 1% ASC. This suggests that while early growth was comparable, iAs exposure attenuates the tumor‐promoting effect of ASCs over time.

### 3.3 Insights Into Immune Cell Infiltration Into Tumors via Computational Deconvolution of RNAseq Data

**FIGURE 3:**
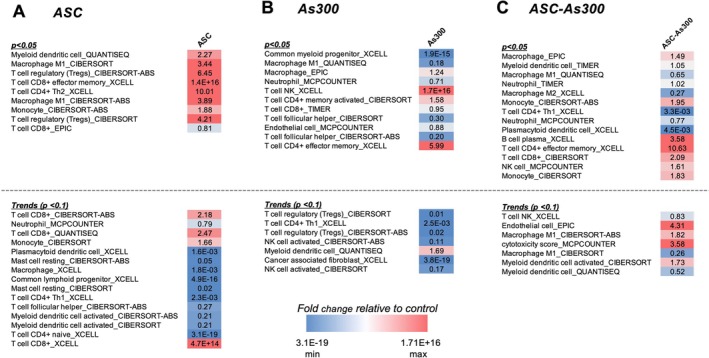
Immune cell composition in the tumor microenvironment inferred from bulk RNA‐seq data using TIMER2.0 deconvolution (Day 21). Heatmap shows the estimated fold‐change in relative abundance of innate and adaptive immune cell signatures compared to control (color bar: red = increase, blue = decrease).

To analyze immune cell composition in the TME, TIMER2.0 was applied to bulk RNAseq data (d21) using multiple deconvolution methods (Figure 3). ASC treatment alone significantly altered TC2Ras tumor immunity, notably reducing pro‐inflammatory M1 macrophages, myeloid dendritic cells, and activated mast cells (*p* < 0.05), indicating suppression of innate immune responses. Transcriptomic signatures inferred included significant increases in regulatory T cells (Tregs), but in contrast, T follicular helper (Tfh) cells were estimated to be reduced, suggesting a shift toward immune suppression. Conversely, CD4+ Th1 and CD8+ effector memory T cell scores were significantly increased, suggesting compensatory memory responses.

The analyses from combination treatment samples (ASC_As300) suggested an enhanced immunosuppression, where signatures of M1/M2 macrophages, neutrophils, CD8+ T cells, and Th1 cells were estimated as reduced. Tregs and plasma B cells scores were elevated, suggesting that the TME may be shifted toward tolerance and humoral immunity. Natural Killer (NK) cells and monocyte signatures were also reduced, suggesting broad innate immune suppression. As300 alone was associated with reduced innate immunity scores inferred, with lower M1 macrophage and neutrophil signatures and a trend toward fewer activated NKs (Figure 3). Adaptive immunity estimates indicated decreased Tfh and CD8^+^ T cell signatures and a pattern of increased Tregs, NKT cells, and CD4^+^ memory T cells. These results would be consistent with a more tolerogenic and chronically activated immune state. Additionally, inferred increases in common myeloid progenitors and cancer‐associated fibroblasts suggested remodeling toward a more immune‐suppressive TME.

### 3.4 Flow Cytometry Analysis of the TME Cellular Composition

**FIGURE 4:**
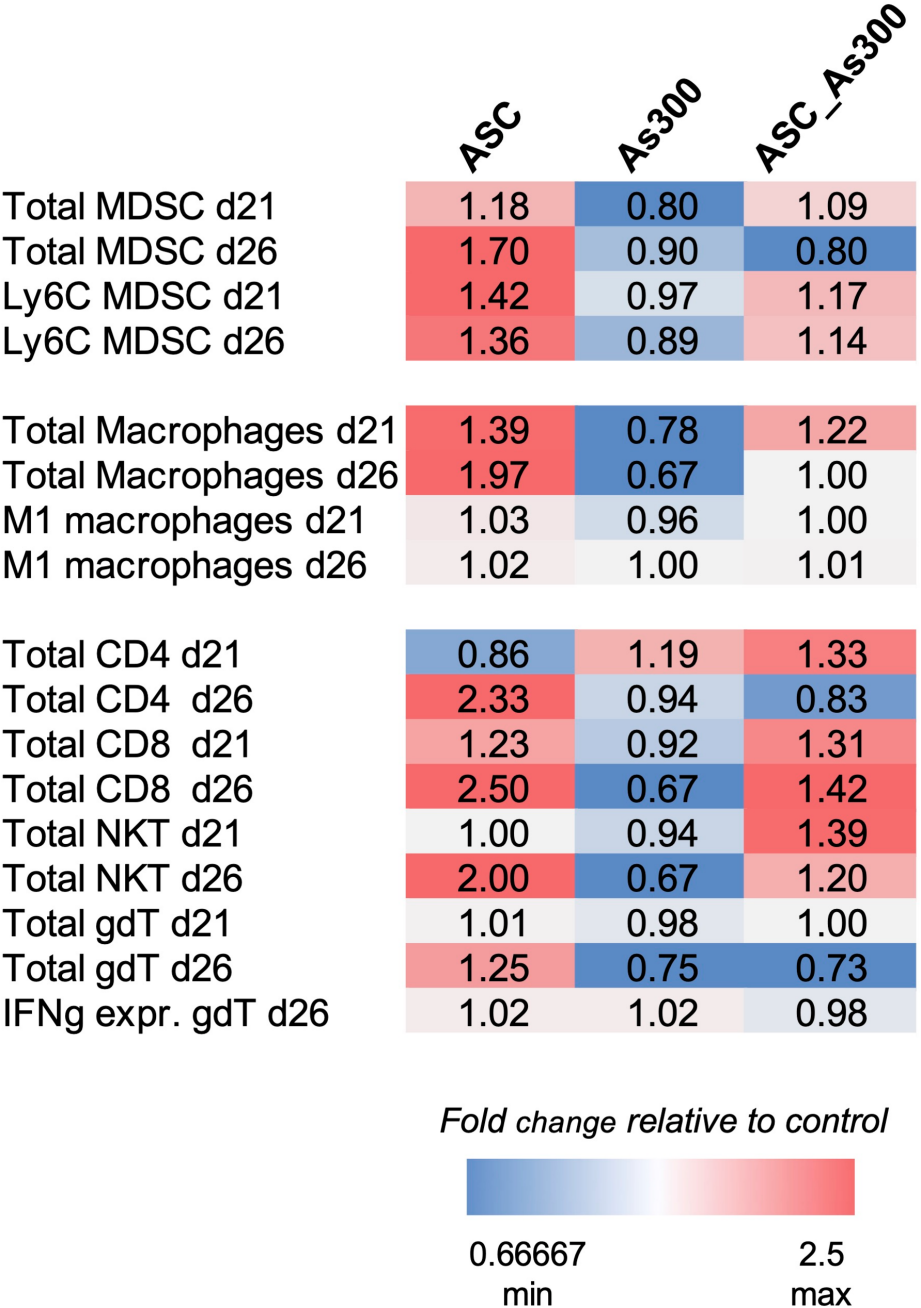
Heatmap Summary of Flow Cytometry Data for Myeloid and T Cell Infiltration in Tumors. (A) *Myeloid‐Derived Suppressor Cells (MDSCs).* Total MDSCs (CD11b^+^Gr‐1^+^) and monocytic MDSCs (Ly6C^+^ subset) were quantified at days 21 and 26 posttumor implantation. (B*) Macrophages*. Total macrophages (CD11b^+^F4/80^+^) and M1 phenotype (CD38^+^ subset) were assessed at days 21 and 26. (C) *Tumor‐Infiltrating Lymphocytes*. CD4^+^ T helper cells, CD8^+^ cytotoxic T cells, and NKT cells (TCR Vβ7^+^) were quantified at days 21 and 26. (D) *γδ T Cells and IFN*γ *Expression*. γδT cells (TCR γ/δ^+^) were measured at Days 21 and 26; intracellular IFNγ expression was assessed at Day 26. Data are normalized to control (value = 1), shown as mean ± SD Statistical significance (*p < 0.05) was determined by two‐way ANOVA.

To assess iAs exposure and ASC co‐implantation effects on immune cell recruitment in the TME, flow cytometry was performed on excised tumors at Days 21 and 26, focusing on MDSCs, macrophages, and T lymphocytes (including γδT cells) (Figure 4). MDSCs (CD11b + Gr‐1+) levels were modestly raised in the ASC group at Day 21 (1.18‐fold vs. control) and significantly increased in As300 at Day 26 (1.7‐fold), while ASC_As300 showed reduced recruitment (0.80‐fold), suggesting that ASC co‐treatment attenuates iAs‐induced MDSC expansion. Ly6C was used to characterize M‐MDSCs, which were most enriched in ASC at both timepoints (1.42‐ and 1.36‐fold), consistent with ASC‐mediated monocytic infiltration. As300 had lower Ly6C + MDSCs, with ASC_As300 partially restoring this population, showing a complex interaction of iAs and ASC effects.

Macrophages (CD11b+F4/80+) were elevated in ASC at Day 21 (1.4‐fold), reduced in As300 (0.8‐fold) and partly restored in ASC_As300 (1.2‐fold). At Day 26, ASC had the highest (twofold) and As300 the lowest (0.7‐fold) levels, indicating iAs suppresses macrophage infiltration, countered by ASCs. CD38+ (M1‐like) macrophage proportions remained high and unchanged across groups and time, signifying stable polarization (Figure 4). Tumor‐infiltrating lymphocytes were analyzed: CD4+ T cells were slightly reduced in ASC at Day 21 (0.9‐fold), increased in ASC_As300 (1.3‐fold), and most abundant in ASC at Day 26 (2.3‐fold), showing robust delayed recruitment. CD8+ T cells increased in ASC_As300 at Day 21 (1.3‐fold) and in As300 at Day 26 (2.5‐fold), suggesting that iAs boosts late cytotoxic T‐cell recruitment. NKT (TCR Vβ7+) cells rose in ASC_As300 at Day 21 (1.4‐fold) and ASC at Day 26 (2‐fold), so ASC supports innate‐like lymphocyte infiltration, modulated by iAs. γδT cell (TCR γ/δ+) levels stayed steady across groups, and intracellular IFNγ remained high in γδT cells at Day 26 in all, suggesting functional activation without treatment effect.

### 3.5 Global Transcriptomic Analysis and Pathway Analysis of the TME

**FIGURE 5:**
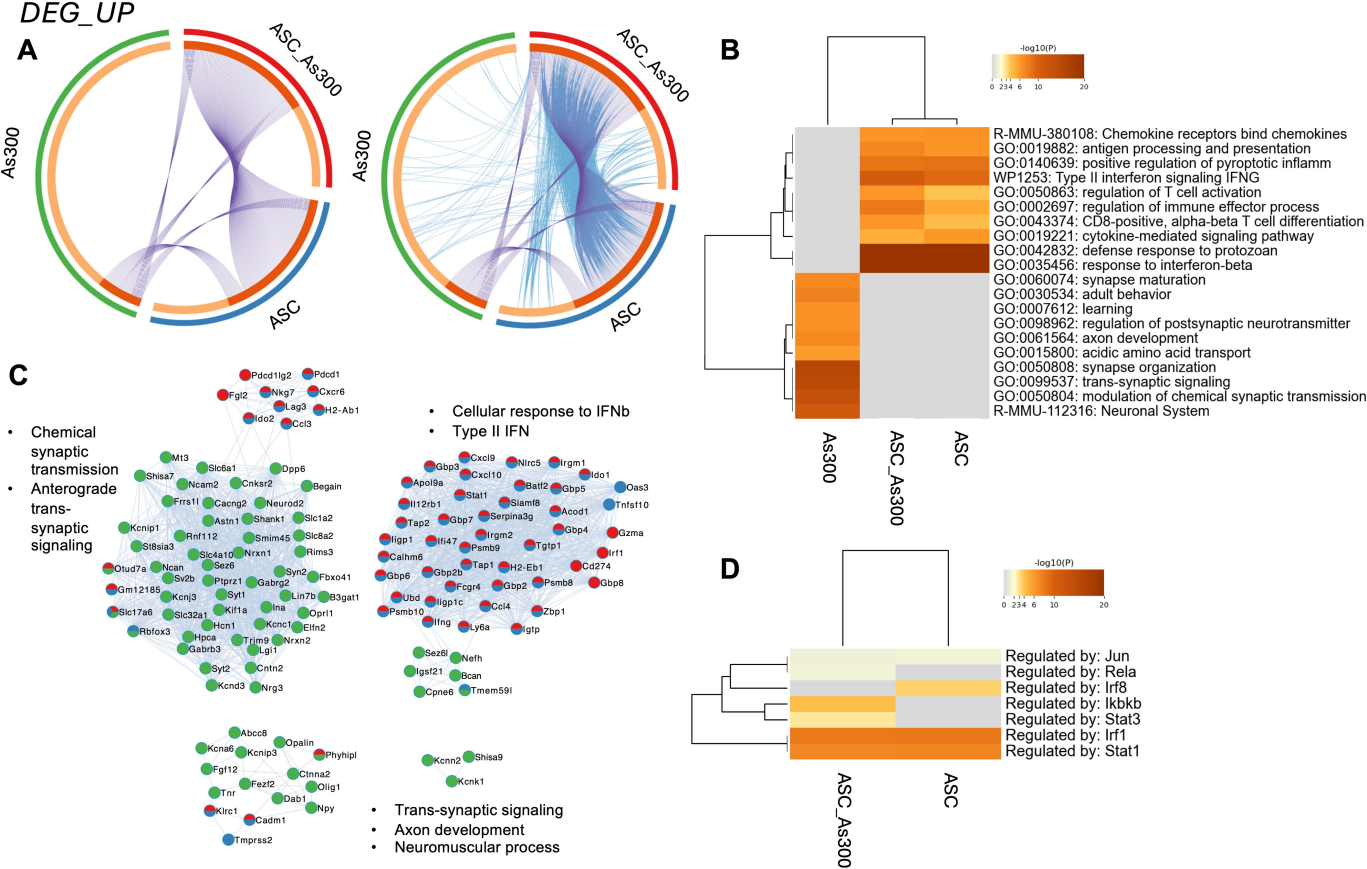
RNA‐seq analysis of differentially expressed genes upregulated (DEG_UP) in TC2Ras tumors at Day 21. (A) Metascape shows ASC groups share the most upregulated genes (left) and enriched biological processes (right). (B) Heatmap of significantly enriched pathways (*p* < 0.05); color intensity reflects –log10 (*p*‐value). (C) Cytoscape network of top MCODE‐identified pathways; node colors: As300 (green), ASC (blue), ASC_As300 (red). (D) TTRUST transcription factor enrichment via Metascape; significant factors were enriched only in ASC and ASC_As300. Color intensity reflects –log_10_ (*p*‐value).

Pathway enrichment analysis from the RNAseq data was conducted to characterize the biological and immune signaling pathways altered by treatment. Metascape analysis showed substantial gene overlap between the *upregulated* DEGs of ASC and ASC_As300 groups (Figure 5A, left panel), as well as shared biological processes (Figure 5B, right panel). Pathways with a network size ≥ 3 genes and *p* < 0.05 included synapse organization and cell–cell junction/transmission in the As300 group (Figure 5C) and several immune‐related pathways common to both ASC and ASC_As300 groups. The ASC_As300 group, which had smaller tumors, showed stronger enrichment (higher –log10(p) values) for pathways including CD8+ T cell differentiation, reduction of immune effector processes, Type II IFN signaling (IFNγ), and antigen processing/presentation. In contrast, the ASC group showed a more significant enrichment for cytokine‐mediated signaling.

Genes involved in these pathways, visualized in the *Cytoscape* network (Figure 5C), included those clustering as anterograde/transsynaptic signaling, with some also linked to PCa. The As300 group had distinct gene clusters compared to ASC groups, which included more immune checkpoint‐related genes (Pdcd1, Cxcr6, Ccl3, and Lag3). The second network included response to IFNβ and Type II IFN, also showing some group‐specific gene sets, with ASC groups including CD274 (PD‐L1). The third network has annotations mostly as synaptic signaling/neuromuscular processes and included genes such as Tmprss2 (ASC), Klrc (both ASC groups), and Fgf12 (As300), some of which are also relevant to PCa. Interestingly, TTRUST transcription factor enrichment (Figure 5D) indicated a significant enrichment of Stat3, Ikbkb, and Rela in genes found to be differentially upregulated in ASC_As300, while Irf8 was uniquely enriched in the ASC group. The As300 group did not yield any significant transcription factor enrichments.

**FIGURE 6:**
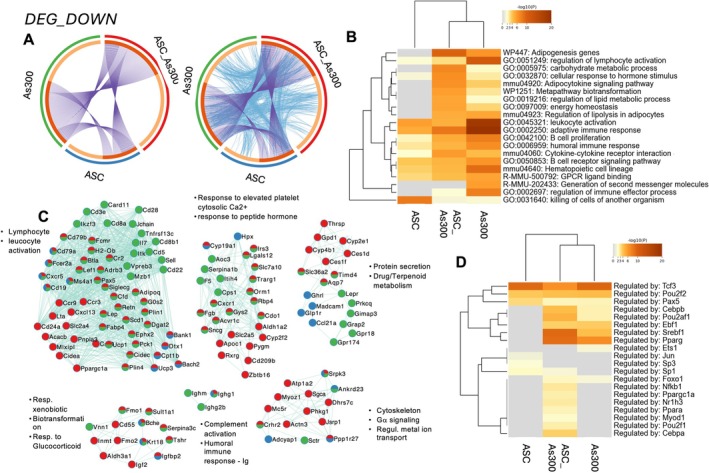
RNA‐seq analysis of downregulated DEGs in TC2Ras tumors at Day 21. (A) Metascape shows ASC groups share the most downregulated genes (left) and enriched biological processes (right). (B) Heatmap of significantly enriched pathways (*p* < 0.05) across tumor groups; color intensity reflects –log10 (*p*‐value). (C) Cytoscape network of top MCODE‐identified pathways; node colors: As300 (green), ASC (blue), ASC_As300 (red). (D) TTRUST transcription factor enrichment via Metascape. Color intensity reflects –log_10_ (*p*‐value).

Pathway enrichment analysis of *downregulated DEGs* revealed the biological processes suppressed by treatment across ASC, ASC_As300, and As300 groups (Figure 6A). Metascape analysis indicated a substantial overlap in downregulated genes between ASC_As300 and As300 groups, with As300 showing the strongest enrichment for immune‐related processes (Figure 6B). Specifically, pathways with network size ≥ 3 genes and *p* < 0.05 included lymphocyte activation, leukocyte activation, adaptive immune response, hematopoietic cell lineage, and regulation of immune effector processes, all markedly reduced in As300 tumors. In contrast, ASC_As300 downregulated pathways were primarily metabolic and signaling‐related, including adipogenesis, carbohydrate metabolism, lipid metabolic process and lipolysis, cytokine‐cytokine receptor interaction, and cellular response to hormone stimulus. The ASC group exhibited fewer unique downregulated processes, with the most significant being killing of cells of another organism, while sharing some immune‐related suppression with the other groups but lacking the metabolic components.

Cytoscape network visualization (Figure 6C) highlighted key clusters of downregulated genes. The largest network corresponded to lymphocyte/leukocyte activation, followed by response to elevated platelet cytosolic Ca^2+^/response to peptide hormone, and a third cluster involving protein secretion/drug/terpenoid metabolism. Additional networks included cytoskeleton organization, Gα signaling, regulation of metal ion transport, and smaller clusters such as response to xenobiotic stimulus, biotransformation, glucocorticoid response, and a three‐member network for complement activation/humoral immune response. Transcription factor enrichment analysis using TRRUST (Figure 6D) showed some regulatory signatures among downregulated genes. The As300 group showed strong enrichment for Pparγ, Tcf3, Ebf1, Srebp1, and Pou2f2, suggesting suppression of lipid metabolism and B‐cell lineage programs. ASC_As300 exhibited enrichment for Tcf3, Cebp, Pou2af1, Ebf1, and Cebpa, with particularly strong signals for Srebp1 and Pparγ, consistent with metabolic pathway downregulation. The ASC group was characterized by Tcf3 and Pou2f2, with higher Pax5 enrichment compared to other groups, indicating selective repression of B‐cell differentiation pathways.

### 3.6 Targeted Gene Expression Validation

**FIGURE 7:**
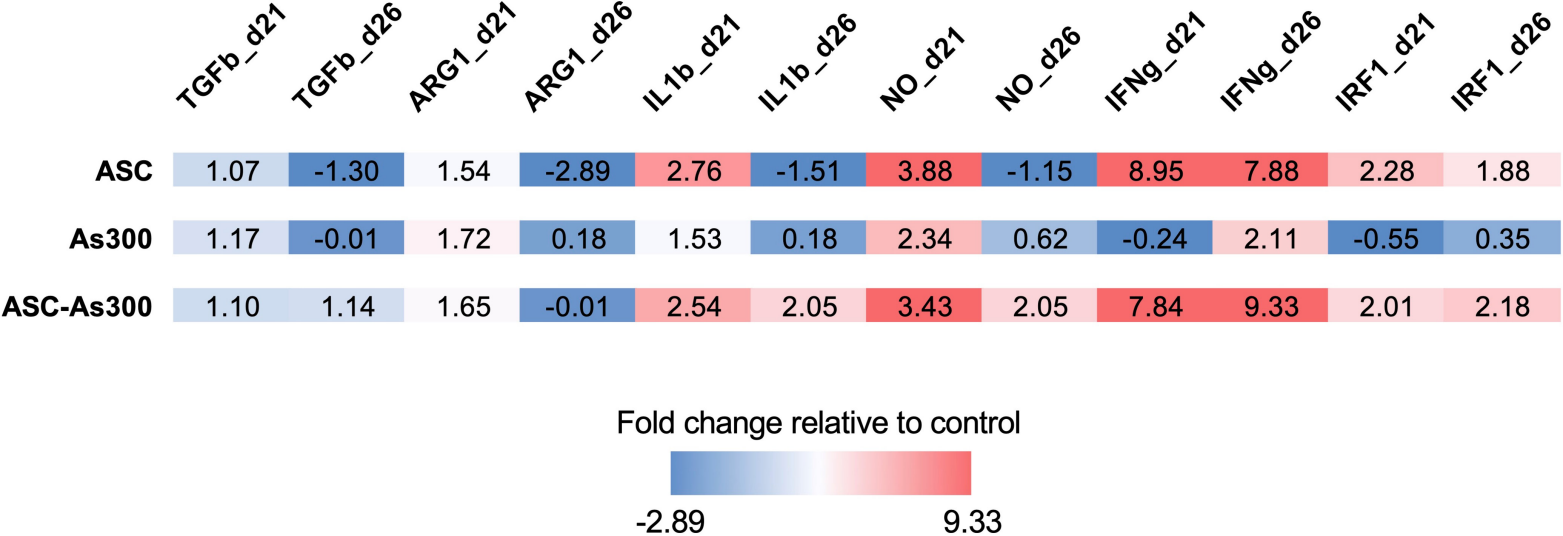
Heatmap of gene expression changes validated by RT‐qPCR. Fold changes in expression of immune‐related genes (TGFβ, Arg1, IL‐1β, iNOS, IFNγ, IRF1) in TC2Ras tumors at Days 21 and 26 across ASC, As300, and ASC_As300 groups. Color intensity reflects relative expression compared to control (0 ppb iAs + 0% ASC).

Given the immune processes revealed by RNAseq, we validated selected findings via qRT‐PCR, confirming strong upregulation of IFNγ and its downstream effector IRF1 in tumors (Figure 7). Genes selected for validation also included IL‐1β, iNOS, Arg1, and TGFβ, based on their relevance to inflammation and immune modulation. These findings were consistent across both time points, suggesting that a potential shift may have occurred in the TME's immune modulatory activity. Additionally, genes associated with myeloid cell activation and inflammation, such as IL‐1β and iNOS, were elevated in ASC‐containing tumors at 21 days; this effect, however, diminished by Day 26. TGFβ expression remained within ± 1.5‐fold across all groups and timepoints (Figure 7), indicating no significant modulation. Arg1 showed a modest upregulation (1.5–1.7 fold) at Day 21 in all groups compared to control untreated, yet by Day 26, Arg1 expression declined in both ASC and ASC_As300 groups but was maintained in As300 tumors at a low level. IL‐1β and iNOS exhibited parallel expression patterns, consistent with known regulatory interactions. At Day 21, ASC and ASC_As300 tumors showed elevated expression of both genes, while As300 tumors showed lower induction. By Day 26, expression of both genes declined in ASC tumors, remained low in As300, and was sustained in ASC_As300 tumors, suggesting a transient inflammatory response primarily driven by ASC. IFNγ expression was markedly elevated in tumors containing ASC, regardless of iAs exposure. At Day 21, ASC and ASC_As300 tumors showed 8‐ to 9‐fold increases, while As300 tumors showed no induction. This upregulation persisted at Day 26, indicating a sustained IFNγ‐driven inflammatory tone in ASC‐containing tumors. IRF1, a downstream effector, mirrored IFNγ expression trends with significant upregulation in ASC and ASC_As300 tumors at both time points. As300 tumors showed no significant IRF1 induction. Together, these results suggest that ASC presence in tumors drives a robust and sustained IFNγ‐IRF1 axis activation, independent of iAs exposure, and is accompanied by transient increases in IL‐1β and iNOS expression. These findings support a shift in the inflammatory tone of the TME that is mediated by ASC.

## 4 Discussion

A major role of the immune system in cancer is to detect and eliminate transformed cells before they evolve mechanisms of immune evasion (Swann and Smyth 2007). Yet, both immune and stromal cells within the TME can paradoxically promote or inhibit tumor growth (de Visser et al. 2006; Erasha et al. 2025). MSCs, enriched in the TME, similarly can engage in broad immune crosstalk (Tyndall 2014) and exert context‐dependent effects. In the present work, we show that ASC‐conditioned media enhances PCa cell viability in vitro, an effect attenuated by iAs exposure. In vivo, ASC co‐implantation increased tumor burden, while iAs altered immune cell recruitment and gene expression. Flow cytometry and RNA‐seq deconvolution suggested that ASCs and iAs may modulate both the innate and adaptive immune compartments, thus driving immune suppression, compensatory memory responses, and shifts in macrophage/MDSC dynamics. The mechanisms appear to involve sustained IFNγ‐IRF1 activation in ASC‐containing tumors, alongside transient inflammatory signatures and downregulated adaptive immunity in iAs‐exposed tumors, as shown by transcriptomics and qPCR analyses. These combined findings suggest that ASCs and iAs converge to reshape the TME through distinct yet overlapping mechanisms, influencing tumor growth, immune infiltration, and inflammatory tone.

Our in vitro results showed that ASCs increased PCa cell viability, an effect modulated by iAs exposure, indicating that ASCs may be sensitive to iAs. While the use of ASC‐CM is useful for probing soluble signaling, it does not capture essential features of the in vivo TME, including direct cell–cell contact, ECM cues, and spatial organization that may shape stromal, immune, and tumor interactions. Thus, although CM may partially reflect the influence of ASC; complementary 3D co‐cultures or in vivo models may be necessary to fully define how ASC modulate tumor‐immune interactions. While iAs is regarded widely as immunotoxic (Dangleben et al. 2013), its impact within the TME has remained unclear. Therefore, we pursued two questions: (1) How might ASCs influence immune cell recruitment and tumor growth in the TME? and (2) In what ways might iAs exposure modify these outcomes? Given the fundamental role of immunity in tumor control, we hypothesized that ASCs and iAs could influence tumor burden via effects on immune cell dynamics. Tumor weights showed ASC co‐mixing (without iAs) increased mass, consistent with reports of MSC‐driven PCa enhancement, and context‐dependent effects (Cheng et al. 2016; Luo et al. 2015; Wen et al. 2015).

The ASC‐CM‐induced increase in TC2Ras viability is consistent with known ASC‐secreted factors such as IL‐6 family cytokines, IGF‐1, FGF/EGF ligands, TGF‐β, CXCL12, and SPP1, which can promote tumor growth and modulate immune pathways. Several of these pathways overlap with our RNA‐seq immune signatures, suggesting coordinated proliferative and immunomodulatory effects. Targeted depletion or neutralization studies would be needed to identify the key mediators. Nevertheless, iAs reversed this trend, suggesting that environmental factors can alter stromal influence. And while some studies show that MSCs can suppress cancer growth using CM (Lee et al. 2015), naïve (Takahara et al. 2014), or engineered (Zolochevska et al. 2012) ASCs, there remains debate on whether MSCs are ultimately pro‐ or anti‐tumorigenic (Rivera‐Cruz and Figueiredo 2023; Rivera‐Cruz et al. 2023; Rivera‐Cruz et al. 2017). In the 26‐day groups, absent ASC, 300 ppb of iAs did not affect tumor size, so this dose does not simply kill cells. However, in ASC‐mixed tumors, iAs led to a notable size reduction, indicating that iAs can negate the ASC‐mediated promotion of growth, consistent with in vitro data on ASC‐GFP CM and TC2Ras cells. Previously, iAs was shown to switch human ASC secretomes from anti‐ to pro‐proliferative (Shearer et al. 2016), yet our in vivo results contrast: ASC increases tumor size and iAs attenuates it. Our 1‐week iAs exposure may model soluble environmental stress but does not fully reflect long‐term, low‐dose human exposure, which can induce cumulative, epigenetic, and immunologic changes. Prolonged exposure also may result in distinct ASC or immune phenotypes relative to short‐term treatments. Future studies using extended or stepwise chronic iAs exposure may help determine how environmentally‐relevant dosing may shape ASC function and tumor‐immune interactions. This may result from complex crosstalk and experimental variations such as ASC species, PCa model, and systemic iAs exposure, each influencing cytokine milieu and proliferation.

Tumor size evaluation alone is insufficient; thus, we also examined several immune cell populations. including MDSCs, which are immunosuppressive (Umansky et al. 2019; Umansky et al. 2016). Flow cytometry showed that total MDSC recruitment was similar between groups, apart from a minor increase with ASCs at Day 21 and a clear drop in the ASC_As300 group at Day 26, with the lowest in iAs‐only tumors. Unexposed ASC‐mixed tumors showed increased M‐MDSC, implicated in soluble factor‐driven suppression, unlike ROS‐dependent G‐MDSC (Youn and Gabrilovich 2010); this may strongly modulate TME signaling and tumor progression, correlating with reports in multiple myeloma (Wang et al. 2015). Though TIMER2.0 does not single out MDSCs, it does estimate proportions of monocytes and M2 macrophages. Monocytes (M‐MDSC progenitors) were highest in ASC tumors, mirroring flow cytometry findings, while iAs diminished monocyte and M2 macrophage signatures, suggesting curtailed immunosuppressive recruitment. We further examined ASC and iAs effects on macrophage infiltration. Early (Day 21), ASC co‐mixing elevated macrophage recruitment regardless of iAs, but this was reversed by Day 26 after iAs exposure. MSC‐driven factors may underlie this effect, as seen in wound healing models (Chen et al. 2008). In tumors, MSCs preferentially recruit M2 macrophages (Ren et al. 2012), though our ASC‐GFP cells were not tumor‐derived. Notably, > 90% of recruited macrophages were M1‐type, which are typically antitumorigenic. This pattern, with M1 prevalence in confined disease and M2 in advanced (Lanciotti et al. 2014), is echoed in our TIMER2.0 data and may indicate that a less‐aggressive TME is induced by ASC, and disrupted by iAs.

The suppressive effect of iAs on macrophage recruitment could result from altered ASC cytokine profiles neutralizing immune‐stimulatory outputs or from systemic iAs prompting chronic inflammation that hinders macrophage homing. Similar innate immune blunting following iAs has been seen in zebrafish (Nayak et al. 2007). CD4+ T cells, known macrophage activators (Mosser and Edwards 2008), could also shape M1 abundance in the TME, and our findings suggest that iAs exposure dampens immune cell recruitment by ASCs over time. Cell–cell signaling in the TME is a network response. IFNγ and downstream IRF1 were significantly increased with ASC; since IFNγ drives immune activation, with cross‐talk between macrophages and lymphocytes instigating M1 polarization (Biswas and Mantovani 2010), it can also aid in tumorigenesis (Zaidi 2019) and thus is implicated in M‐MDSC‐mediated suppression (Movahedi et al. 2008). This dual IFNγ role might explain concurrent protumor and antitumor responses in ASC‐commixed groups. No overall IFNγ change followed iAs, but past work with human ASCs (Shearer et al. 2016) and in exposed populations (Biswas et al. 2008) shows a concentration‐dependent IFNγ decrease, potentially explaining immune suppression with iAs.

Furthermore, RNA‐seq analyses revealed enrichment of pathways related to CD8+ T cell differentiation, type II IFN signaling, and antigen processing/presentation in ASC_As300 tumors, which were smaller in size and showed stronger immune activation signatures. Immune checkpoint genes such as Pdcd1, Lag3, and Cd274 were also upregulated, suggesting a compensatory inhibitory axis that may balance effector responses. Synapse organization and cell–cell junction pathways might further suggest enhanced immune–tumor interactions, while transcription factor enrichment for Stat3, Ikbkb, and Rela in ASC_As300 tumors aligns with sustained inflammatory signaling. In contrast, downregulated networks indicated instead a broad suppression of adaptive immunity and metabolic programs in the iAs‐exposed tumor samples. Meanwhile, the As300 group showed the strongest reductions in pathways relating to lymphocyte activation, leukocyte activation, hematopoietic lineage, and ASC_As300 tumors downregulated pathways relating to adipogenesis, lipid metabolism, and cytokine/receptor interactions. Transcription factor analysis pointed to pparg and Srebf1 as key regulators of these suppressed metabolic processes, alongside Tcf3 and Pou2f2 (which govern B‐cell lineage commitment). These findings suggest that iAs exposure not only blunts immune effector pathways but also can rewire TME metabolic circuits, thus constraining immune cell function and tumor‐stromal crosstalk. Together, the transcriptomic insights reinforce the duality of ASC when exposed to iAs‐ASC can on the one hand promote immune activation and inflammatory tone, yet iAs imposes broad immunosuppressive and metabolic constraints. For instance, immune profiling data pointed to ASC promoting tumor progression via immune modulation, with iAs exposure countering this by depleting key pro‐inflammatory and cytotoxic cells. For example, the reduced M1, Th1, and CD8+ T numbers in ASC + iAs tumors may suggest a loss of cytotoxic control and IFNγ signaling. Tregs and plasma B cells were estimated as increased in both ASC and ASC + iAs groups, indicating potential immunosuppressive and humoral skewing, along with memory T‐cell increases, signaling a possible shift away from effector function. This analysis suggested increased myeloid progenitors and fibroblasts in the iAs group, indicative of stromal remodeling and further immune escape. Thus, ASC may drive initial immune activation, while iAs exposure inhibits this, promoting a TME that is permissive to tumors. Interestingly, few GFP+ ASC cells remain detectable within tumors at late timepoints (< 0.1%), possibly due to apoptosis, host cell expansion, or GFP loss; still, their early presence drives lasting changes to TME and immune composition.

This study can serve to highlight the critical influence that stromal and environmental factors can play on the immune dynamics within the TME. While immune cell immunophenotyping does not fully capture functional states and the use of mouse models and experimentally added ASCs introduces constraints on generalizability, these approaches were essential to this study to enable us to examine these complex interactions in an immunocompetent setting. The observed effects of ASCs and iAs on immune composition and inflammatory composition of the TME reveal mechanisms that merit deeper investigation. These findings may provide a foundation for future work aimed at refining targeted immunotherapies and informing public health as it relates to environmental exposures.

## 5 Conclusions

In summary, ASCs can promote prostate tumor growth and modulate immune composition, while iAs imposes immunosuppressive and metabolic constraints that counter the ASC‐driven activation. These findings highlight the importance of studying how stromal‐environmental interactions shape the TME and uncover several future directions for identifying key ASC mediators and defining chronic iAs effects, potentially leading to more informed therapeutic and public health strategies.

## Author Contributions

J.S., C.R.‐C., A.M.B.C., and M.L.F. contributed to methodology, writing, and editing. J.S. and M.L.F. performed data collection. C.E.F.‐A. contributed to writing and editing.

## Funding

This work was supported in part by the Purdue University Center for Cancer Research, NIH grant P30 CA023168.

## Ethics Statement

The authors have nothing to report.

## Consent

The authors have nothing to report.

## Conflicts of Interest

The authors declare no conflicts of interest.

## Animal Studies

All animal procedures were approved by the Institutional Animal Care and Use Committee (IACUC) at Purdue University and carried out according to the guidelines for the care and use of laboratory animals.

## Supporting information


**Table S1:** List of antibodies used for flow cytometry.

## Data Availability

The RNAseq data for this submission are available at NCBI GEO record GSE317208.

## References

[jat70108-bib-0001] Alvarado‐Morales, I. , V. Olivares‐Illana , C. Arenas‐Huertero , et al. 2021. “Human Prostate Epithelial Cells and Prostate‐Derived Stem Cells Malignantly Transformed In Vitro With Sodium Arsenite Show Impaired Toll Like Receptor −3 (TLR3)‐Associated Anti‐Tumor Pathway.” Toxicology Letters 350: 185–193. 10.1016/j.toxlet.2021.07.013. 34303791 PMC8410676

[jat70108-bib-0002] Biswas, R. , P. Ghosh , N. Banerjee , et al. 2008. “Analysis of T‐Cell Proliferation and Cytokine Secretion in the Individuals Exposed to Arsenic.” Human & Experimental Toxicology 27: 381–386. 10.1177/0960327108094607. 18715884

[jat70108-bib-0003] Biswas, S. K. , and A. Mantovani . 2010. “Macrophage Plasticity and Interaction With Lymphocyte Subsets: Cancer as a Paradigm.” Nature Immunology 11: 889–896. 10.1038/ni.1937. 20856220

[jat70108-bib-0004] Bommarito, P. A. , R. Beck , C. Douillet , et al. 2019. “Evaluation of Plasma Arsenicals as Potential Biomarkers of Exposure to Inorganic Arsenic.” Journal of Exposure Science & Environmental Epidemiology 29: 718–729. 10.1038/s41370-019-0121-x. 30728485 PMC6684877

[jat70108-bib-0005] Brennen, W. N. , S. R. Denmeade , and J. T. Isaacs . 2013. “Mesenchymal Stem Cells as a Vector for the Inflammatory Prostate Microenvironment.” Endocrine‐Related Cancer 20: R269–R290. 10.1530/ERC-13-0151. 23975882 PMC3994592

[jat70108-bib-0006] Brennen, W. N. , L. N. Kisteman , and J. T. Isaacs . 2016. “Rapid Selection of Mesenchymal Stem and Progenitor Cells in Primary Prostate Stromal Cultures.” Prostate 76: 552–564. 10.1002/pros.23145. 26732992 PMC4856028

[jat70108-bib-0007] Buus, T. B. , M. H. Jee , and N. Ødum . 2019. “OMIP‐057: Mouse γδ T‐Cell Development Characterized by a 14 Color Flow Cytometry Panel.” Cytometry. Part A 95: 726–729. 10.1002/cyto.a.23754. 30977950

[jat70108-bib-0008] Chen, L. , E. E. Tredget , P. Y. G. Wu , and Y. Wu . 2008. “Paracrine Factors of Mesenchymal Stem Cells Recruit Macrophages and Endothelial Lineage Cells and Enhance Wound Healing.” PLoS ONE 3: e1886. 10.1371/journal.pone.0001886. 18382669 PMC2270908

[jat70108-bib-0009] Cheng, J. , K. Yang , Q. Zhang , et al. 2016. “The Role of Mesenchymal Stem Cells in Promoting the Transformation of Androgen‐Dependent Human Prostate Cancer Cells Into Androgen‐Independent Manner.” Scientific Reports 6: 16993. 10.1038/srep16993. 26787499 PMC4726385

[jat70108-bib-0010] Cuiffo, B. G. , and A. E. Karnoub . 2012. “Mesenchymal Stem Cells in Tumor Development: Emerging Roles and Concepts.” Cell Adhesion & Migration 6: 220–230. 10.4161/cam.20875. 22863739 PMC3427236

[jat70108-bib-0011] Dangleben, N. L. , C. F. Skibola , and M. T. Smith . 2013. “Arsenic Immunotoxicity: A Review.” Environmental Health 12: 73. 10.1186/1476-069X-12-73. 24004508 PMC3848751

[jat70108-bib-0012] de Visser, K. E. , A. Eichten , and L. M. Coussens . 2006. “Paradoxical Roles of the Immune System During Cancer Development.” Nature Reviews. Cancer 6: 24–37. 10.1038/nrc1782. 16397525

[jat70108-bib-0013] Du, M. , L. Sun , J. Guo , and H. Lv . 2024. “Macrophages and Tumor‐Associated Macrophages in the Senescent Microenvironment: From Immunosuppressive TME to Targeted Tumor Therapy.” Pharmacological Research 204: 107198. 10.1016/j.phrs.2024.107198. 38692466

[jat70108-bib-0014] Erasha, A. M. , H. El‐Gendy , A. S. Aly , M. Fernández‐Ortiz , and R. K. A. Sayed . 2025. “The Role of the Tumor Microenvironment (TME) in Advancing Cancer Therapies: Immune System Interactions, Tumor‐Infiltrating Lymphocytes (TILs), and the Role of Exosomes and Inflammasomes.” International Journal of Molecular Sciences 26. 10.3390/ijms26062716. PMC11942452 40141358

[jat70108-bib-0015] Escudero‐Lourdes, C. , I. Alvarado‐Morales , and E. J. Tokar . 2022. “Stem Cells as Target for Prostate Cancer Therapy: Opportunities and Challenges.” Stem Cell Reviews and Reports 18: 2833–2851. 10.1007/s12015-022-10437-6. 35951166 PMC9716656

[jat70108-bib-0016] Figueiredo, M. L. , R. Letteri , D. Chan‐Seng , S. Kumar , C. M. Rivera‐Cruz , and T. S. Emrick . 2021. “Reengineering Tumor Microenvironment With Sequential Interleukin Delivery.” Bioengineering (Basel) 8. 10.3390/bioengineering8070090. PMC8301035 34209203

[jat70108-bib-0017] Figueiredo, M. L. , S. Utturkar , S. Kumar , and C. E. Fonseca‐Alves . 2024. “Transcriptomic Analysis of Mouse TRAMP Cell Lines and Tumors Provide Insights Into Shared Pathways and Therapeutic Targets.” Cell Insight 3: 100184. 10.1016/j.cellin.2024.100184. 39175940 PMC11339039

[jat70108-bib-0018] Frisbie, S. H. , and E. J. Mitchell . 2022. “Arsenic in Drinking Water: An Analysis of Global Drinking Water Regulations and Recommendations for Updates to Protect Public Health.” PLoS ONE 17: e0263505. 10.1371/journal.pone.0263505. 35385526 PMC8985943

[jat70108-bib-0019] Giraldo, N. A. , R. Sanchez‐Salas , J. D. Peske , et al. 2019. “The Clinical Role of the TME in Solid Cancer.” British Journal of Cancer 120: 45–53. 10.1038/s41416-018-0327-z. 30413828 PMC6325164

[jat70108-bib-0020] Goliwas, K. F. , J. S. Deshane , C. A. Elmets , and M. Athar . 2021. “Moving Immune Therapy Forward Targeting TME.” Physiological Reviews 101: 417–425. 10.1152/physrev.00008.2020. 32790578 PMC8428923

[jat70108-bib-0021] Grotzer, M. A. , R. Patti , B. Geoerger , A. Eggert , T. T. Chou , and P. C. Phillips . 2000. “Biological Stability of RNA Isolated From RNA Later‐Treated Brain Tumor and Neuroblastoma Xenografts.” Medical and Pediatric Oncology 34: 438–442. 10.1002/(sici)1096-911x(200006)34:6<438:aid-mpo12>3.0.co;2-q. 10842254

[jat70108-bib-0022] Höchst, B. , J. Mikulec , T. Baccega , et al. 2015. “Differential Induction of Ly6G and Ly6C Positive Myeloid Derived Suppressor Cells in Chronic Kidney and Liver Inflammation and Fibrosis.” PLoS ONE 10: e0119662. 10.1371/journal.pone.0119662. 25738302 PMC4349817

[jat70108-bib-0023] Hocking, A. M. 2015. “The Role of Chemokines in Mesenchymal Stem Cell Homing to Wounds.” Adv Wound Care (New Rochelle) 4: 623–630. 10.1089/wound.2014.0579. 26543676 PMC4620518

[jat70108-bib-0024] IARC Working Group on the Evaluation of Carcinogenic Risks to Humans . 2012. “Arsenic, Metals, Fibres, and Dusts.” In IARC Monographs on the Evaluation of Carcinogenic Risks to Humans, vol. 100, 11–465. 23189751 PMC4781271

[jat70108-bib-0025] Jablonski, K. A. , S. A. Amici , L. M. Webb , et al. 2015. “Novel Markers to Delineate Murine M1 and M2 Macrophages.” PLoS ONE 10: e0145342. 10.1371/journal.pone.0145342. 26699615 PMC4689374

[jat70108-bib-0026] Kido, L. A. , C. de Almeida Lamas , M. R. Maróstica , and V. H. A. Cagnon . 2019. “Transgenic Adenocarcinoma of the Mouse Prostate (TRAMP) Model: A Good Alternative to Study PCa Progression and Chemoprevention Approaches.” Life Sciences 217: 141–147. 10.1016/j.lfs.2018.12.002. 30528182

[jat70108-bib-0027] Kumar, S. , D. Lim , H. Kothandaraman , et al. 2025. “Exploring Tumor Dynamics and Responses of Prostate Cancer to IL‐27 Based Treatment Combinations Through Biodynamic Imaging and RNA Sequencing Analyses.” Scientific Reports 15: 42265. 10.1038/s41598-025-26300-w. 41298674 PMC12658048

[jat70108-bib-0028] Lanciotti, M. , L. Masieri , M. R. Raspollini , et al. 2014. “The Role of M1 and M2 Macrophages in Prostate Cancer in Relation to Extracapsular Tumor Extension and Biochemical Recurrence After Radical Prostatectomy.” BioMed Research International 2014: 486798. 10.1155/2014/486798. 24738060 PMC3967497

[jat70108-bib-0029] Lee, J.‐H. , C. H. Park , K.‐H. Chun , and S.‐S. Hong . 2015. “Effect of Adipose‐Derived Stem Cell‐Conditioned Medium on the Proliferation and Migration of B16 Melanoma Cells.” Oncology Letters 10: 730–736. 10.3892/ol.2015.3360. 26622561 PMC4508978

[jat70108-bib-0030] Li, T. , J. Fu , Z. Zeng , et al. 2020. “TIMER2.0 for Analysis of Tumor‐Infiltrating Immune Cells.” Nucleic Acids Research 48: W509–W514. 10.1093/nar/gkaa407. 32442275 PMC7319575

[jat70108-bib-0031] Liang, W. , X. Chen , S. Zhang , et al. 2021. “Mesenchymal Stem Cells as a Double‐Edged Sword in Tumor Growth: Focusing on MSC‐Derived Cytokines.” Cellular & Molecular Biology Letters 26: 3. 10.1186/s11658-020-00246-5. 33472580 PMC7818947

[jat70108-bib-0032] Liu, S. , Z. Wang , R. Zhu , F. Wang , Y. Cheng , and Y. Liu . 2021. “Three Differential Expression Analysis Methods for RNA Sequencing: Limma, EdgeR, DESeq2.” Journal of Visualized Experiments. 10.3791/62528. 34605806

[jat70108-bib-0033] Luo, J. , S. O. Lee , Y. Cui , R. Yang , L. Li , and C. Chang . 2015. “Infiltrating Bone Marrow Mesenchymal Stem Cells (BM‐MSCs) Increase Prostate Cancer Cell Invasion via Altering the CCL5/HIF2α/Androgen Receptor Signals.” Oncotarget 6: 27555–27565. 10.18632/oncotarget.4515. 26342197 PMC4695008

[jat70108-bib-0034] Mosser, D. M. , and J. P. Edwards . 2008. “Exploring the Full Spectrum of Macrophage Activation.” Nature Reviews. Immunology 8: 958–969. 10.1038/nri2448. PMC2724991 19029990

[jat70108-bib-0035] Movahedi, K. , M. Guilliams , J. Van den Bossche , et al. 2008. “Identification of Discrete Tumor‐Induced Myeloid‐Derived Suppressor Cell Subpopulations With Distinct T Cell‐Suppressive Activity.” Blood 111: 4233–4244. 10.1182/blood-2007-07-099226. 18272812

[jat70108-bib-0036] Naujokas, M. F. , B. Anderson , H. Ahsan , et al. 2013. “The Broad Scope of Health Effects From Chronic Arsenic Exposure: Update on a Worldwide Public Health Problem.” Environmental Health Perspectives 121: 295–302. 10.1289/ehp.1205875. 23458756 PMC3621177

[jat70108-bib-0037] Nayak, A. S. , C. R. Lage , and C. H. Kim . 2007. “Effects of Low Concentrations of Arsenic on the Innate Immune System of the Zebrafish (*Danio rerio*).” Toxicological Sciences 98: 118–124. 10.1093/toxsci/kfm072. 17400579

[jat70108-bib-0038] Pederzoli, F. , M. Raffo , H. Pakula , F. Ravera , P. V. Nuzzo , and M. Loda . 2023. “Stromal Cells in Prostate Cancer Pathobiology: Friends or Foes?” British Journal of Cancer 128: 930–939. 10.1038/s41416-022-02085-x. 36482187 PMC10006214

[jat70108-bib-0039] Prantl, L. , F. Muehlberg , N. M. Navone , et al. 2010. “Adipose Tissue‐Derived Stem Cells Promote Prostate Tumor Growth.” Prostate 70: 1709–1715. 10.1002/pros.21206. 20564322 PMC4977846

[jat70108-bib-0040] Quail, D. F. , and J. A. Joyce . 2013. “Microenvironmental Regulation of Tumor Progression and Metastasis.” Nature Medicine 19: 1423–1437. 10.1038/nm.3394. PMC3954707 24202395

[jat70108-bib-0041] Reed, B. C. , and M. D. Lane . 1980. “Insulin Receptor Synthesis and Turnover in Differentiating 3T3‐L1 Preadipocytes.” Proc Natl Acad Sci USA 77: 285–289. 10.1073/pnas.77.1.285. 6928620 PMC348254

[jat70108-bib-0042] Ren, G. , X. Zhao , Y. Wang , et al. 2012. “CCR2‐Dependent Recruitment of Macrophages by Tumor‐Educated Mesenchymal Stromal Cells Promotes Tumor Development and Is Mimicked by TNFα.” Cell Stem Cell 11: 812–824. 10.1016/j.stem.2012.08.013. 23168163 PMC3518598

[jat70108-bib-0043] Ribeiro, R. , C. Monteiro , V. Catalán , et al. 2012. “Obesity and Prostate Cancer: Gene Expression Signature of Human Periprostatic Adipose Tissue.” BMC Medicine 10: 108. 10.1186/1741-7015-10-108. 23009291 PMC3523039

[jat70108-bib-0044] Ribeiro, R. , C. Monteiro , R. Silvestre , et al. 2012. “Human Periprostatic White Adipose Tissue Is Rich in Stromal Progenitor Cells and a Potential Source of Prostate Tumor Stroma.” Experimental Biology and Medicine (Maywood, N.J.) 237: 1155–1162. 10.1258/ebm.2012.012131. 23038706

[jat70108-bib-0045] Rivera‐Cruz, C. M. , and M. L. Figueiredo . 2023. “Evaluation of Human Adipose‐Derived Mesenchymal Stromal Cell Toll‐Like Receptor Priming and Effects on Interaction With Prostate Cancer Cells.” Cytotherapy 25: 33–45. 10.1016/j.jcyt.2022.09.009. 36257875

[jat70108-bib-0046] Rivera‐Cruz, C. M. , S. Kumar , and M. L. Figueiredo . 2023. “Poly I:C‐Priming of Adipose‐Derived Mesenchymal Stromal Cells Promotes a Pro‐Tumorigenic Phenotype in an Immunocompetent Mouse Model of Prostate Cancer.” Frontiers in Cell and Developmental Biology 11: 1145421. 10.3389/fcell.2023.1145421. 38078010 PMC10703370

[jat70108-bib-0047] Rivera‐Cruz, C. M. , J. J. Shearer , M. Figueiredo Neto , and M. L. Figueiredo . 2017. “The Immunomodulatory Effects of Mesenchymal Stem Cell Polarization Within the Tumor Microenvironment Niche.” Stem Cells International 2017: 4015039. 10.1155/2017/4015039. 29181035 PMC5664329

[jat70108-bib-0048] Robinson, M. D. , D. J. McCarthy , and G. K. Smyth . 2010. “edgeR: A Bioconductor Package for Differential Expression Analysis of Digital Gene Expression Data.” Bioinformatics 26: 139–140. 10.1093/bioinformatics/btp616. 19910308 PMC2796818

[jat70108-bib-0049] Shearer, J. J. , M. Figueiredo Neto , C. S. Umbaugh , and M. L. Figueiredo . 2017. “In Vivo Exposure to Inorganic Arsenic Alters Differentiation‐Specific Gene Expression of Adipose‐Derived Mesenchymal Stem/Stromal Cells in C57BL/6J Mouse Model.” Toxicological Sciences 157: 172–182. 10.1093/toxsci/kfx026. 28206643 PMC5837658

[jat70108-bib-0050] Shearer, J. J. , E. A. Wold , C. S. Umbaugh , C. F. Lichti , C. L. Nilsson , and M. L. Figueiredo . 2016. “Inorganic Arsenic‐Related Changes in the Stromal Tumor Microenvironment in a Prostate Cancer Cell‐Conditioned Media Model.” Environmental Health Perspectives 124: 1009–1015. 10.1289/ehp.1510090. 26588813 PMC4937864

[jat70108-bib-0051] Smeester, L. , P. A. Bommarito , E. M. Martin , et al. 2017. “Chronic Early Childhood Exposure to Arsenic Is Associated With a TNF‐Mediated Proteomic Signaling Response.” Environmental Toxicology and Pharmacology 52: 183–187. 10.1016/j.etap.2017.04.007. 28433805 PMC5796657

[jat70108-bib-0052] Su, F. , A. C. Daquinag , S. Ahn , et al. 2021. “Progression of Prostate Carcinoma Is Promoted by Adipose Stromal Cell‐Secreted CXCL12 Signaling in Prostate Epithelium.” NPJ Precision Oncology 5: 26. 10.1038/s41698-021-00160-9. 33753872 PMC7985375

[jat70108-bib-0053] Sun, Z. , S. Wang , and R. C. Zhao . 2014. “The Roles of Mesenchymal Stem Cells in Tumor Inflammatory Microenvironment.” Journal of Hematology & Oncology 7: 14. 10.1186/1756-8722-7-14. 24502410 PMC3943443

[jat70108-bib-0054] Swann, J. B. , and M. J. Smyth . 2007. “Immune Surveillance of Tumors.” Journal of Clinical Investigation 117: 1137–1146. 10.1172/JCI31405. 17476343 PMC1857231

[jat70108-bib-0055] Takahara, K. , M. Ii , T. Inamoto , et al. 2014. “Adipose‐Derived Stromal Cells Inhibit Prostate Cancer Cell Proliferation Inducing Apoptosis.” Biochemical and Biophysical Research Communications 446: 1102–1107. 10.1016/j.bbrc.2014.03.080. 24680678

[jat70108-bib-0056] Tung, J. W. , D. R. Parks , W. A. Moore , L. A. Herzenberg , and L. A. Herzenberg . 2004. “New Approaches to Fluorescence Compensation and Visualization of FACS Data.” Clinical Immunology 110: 277–283. 10.1016/j.clim.2003.11.016. 15047205

[jat70108-bib-0057] Tyndall, A. 2014. “Mesenchymal Stem Cell Treatments in Rheumatology: A Glass Half Full?” Nature Reviews Rheumatology 10: 117–124. 10.1038/nrrheum.2013.166. 24217581

[jat70108-bib-0058] Umansky, V. , G. J. Adema , J. Baran , et al. 2019. “Interactions Among Myeloid Regulatory Cells in Cancer.” Cancer Immunology, Immunotherapy 68: 645–660. 10.1007/s00262-018-2200-6. 30003321 PMC11028297

[jat70108-bib-0059] Umansky, V. , C. Blattner , C. Gebhardt , and J. Utikal . 2016. “The Role of Myeloid‐Derived Suppressor Cells (MDSC) in Cancer Progression.” Vaccines (Basel) 4. 10.3390/vaccines4040036. PMC5192356 27827871

[jat70108-bib-0060] Umbaugh, C. S. , A. Diaz‐Quiñones , M. F. Neto , J. J. Shearer , and M. L. Figueiredo . 2018. “A Dock Derived Compound Against Laminin Receptor (37 LR) Exhibits Anti‐Cancer Properties in a Prostate Cancer Cell Line Model.” Oncotarget 9: 5958–5978. 10.18632/oncotarget.23236. 29464047 PMC5814187

[jat70108-bib-0061] Wang, Z. , L. Zhang , H. Wang , et al. 2015. “Tumor‐Induced CD14+HLA‐DR (‐/Low) Myeloid‐Derived Suppressor Cells Correlate With Tumor Progression and Outcome of Therapy in Multiple Myeloma Patients.” Cancer Immunology, Immunotherapy 64: 389–399. 10.1007/s00262-014-1646-4. 25548095 PMC11028624

[jat70108-bib-0062] Wen, S. , Y. Niu , S. Yeh , and C. Chang . 2015. “BM‐MSCs Promote Prostate Cancer Progression via the Conversion of Normal Fibroblasts to Cancer‐Associated Fibroblasts.” International Journal of Oncology 47: 719–727. 10.3892/ijo.2015.3060. 26095189 PMC4501667

[jat70108-bib-0063] Xu, Y. , E. J. Tokar , and J. Pi . 2021. “Arsenic as an Environmental Toxicant and a Therapeutic Agent: Foe and Friend.” Toxicology and Applied Pharmacology 415: 115438. 10.1016/j.taap.2021.115438. 33548274

[jat70108-bib-0064] Youn, J.‐I. , and D. I. Gabrilovich . 2010. “The Biology of Myeloid‐Derived Suppressor Cells: The Blessing and the Curse of Morphological and Functional Heterogeneity.” European Journal of Immunology 40: 2969–2975. 10.1002/eji.201040895. 21061430 PMC3277452

[jat70108-bib-0065] Zaidi, M. R. 2019. “The Interferon‐Gamma Paradox in Cancer.” Journal of Interferon & Cytokine Research 39: 30–38. 10.1089/jir.2018.0087. 30388040 PMC6350411

[jat70108-bib-0066] Zhou, Y. , B. Zhou , L. Pache , et al. 2019. “Metascape Provides a Biologist‐Oriented Resource for the Analysis of Systems‐Level Datasets.” Nature Communications 10: 1523. 10.1038/s41467-019-09234-6. PMC6447622 30944313

[jat70108-bib-0067] Zolochevska, O. , J. Ellis , S. Parelkar , et al. 2013. “Interleukin‐27 Gene Delivery for Modifying Malignant Interactions Between Prostate Tumor and Bone.” Human Gene Therapy 24: 970–981. 10.1089/hum.2013.091. 24028178 PMC3868392

[jat70108-bib-0068] Zolochevska, O. , and M. L. Figueiredo . 2011. “Cell‐Cycle Regulators cdk2ap1 and Bicalutamide Suppress Malignant Biological Interactions Between Prostate Cancer and Bone Cells.” Prostate 71: 353–367. 10.1002/pros.21249. 20812223

[jat70108-bib-0069] Zolochevska, O. , X. Xia , B. J. Williams , A. Ramsay , S. Li , and M. L. Figueiredo . 2011. “Sonoporation Delivery of Interleukin‐27 Gene Therapy Efficiently Reduces Prostate Tumor Cell Growth In Vivo.” Human Gene Therapy 22: 1537–1550. 10.1089/hum.2011.076. 21801027 PMC5915212

[jat70108-bib-0070] Zolochevska, O. , G. Yu , J. M. Gimble , and M. L. Figueiredo . 2012. “Pigment Epithelial‐Derived Factor and Melanoma Differentiation Associated Gene‐7 Cytokine Gene Therapies Delivered by Adipose‐Derived Stromal/Mesenchymal Stem Cells Are Effective in Reducing Prostate Cancer Cell Growth.” Stem Cells and Development 21: 1112–1123. 10.1089/scd.2011.0247. 21671747

