# Supplementary material for: Immunomodulation of the Prostate Tumor Microenvironment Following Inorganic Arsenic Exposure
Source: J Appl Toxicol. 2026 Feb 23;46(9):3274–87. doi: 10.1002/jat.70108 (PMC13432709; doi:10.1002/jat.70108)
Supplement: Supplementary file 1 — Table S1: List of antibodies used for flow cytometry. [file JAT-46-3274-s001.docx]

**Supplementary Table 1.** List of Antibodies Used For Flow Cytometry

| CELL TYPE | GOAL | MARKER | ANTIBODY | COMPANY | CATALOG | CONCENTRATION PER 10^6^ CELLS (µg/µL) |  |
| --- | --- | --- | --- | --- | --- | --- | --- |
| T Cells | T_helper_ | CD4 | PE/Dazzle™ 594 anti-mouse CD4 | Biolegend | 100455 | 0.25 µg |  |
|  | T_cytotoxic_ | CD8 | PerCP/Cy5.5 anti-mouse CD8a | Biolegend | 100734 | 1.0 µg |  |
|  | Gamma-Delta | TCR γ/δ | PE/Cy7 anti-mouse TCR γ/δ | Biolegend | 118123 | 0.25 µg |  |
|  | NKT | TCR Vβ7 | PE anti-mouse TCR Vβ7 | Biolegend | 118307 | 0.25 µg |  |
|  | IFN**γ** | IFN**γ** | APC anti-mouse IFN-**γ** | Biolegend | 505810 | 1.0 µg |  |
| Macrophages | Pan Macrophages | F4/80 | PE/Dazzle™ 594 anti-mouse F4/80 | Biolegend | 123145 | 0.5 µg |  |
|  |  | CD11b | PE/Cy7 anti-mouse CD11b | Biolegend | 101215 | 0.25 µg |  |
|  | M1 | CD38 | PE anti-mouse CD38 | Biolegend | 102707 | 0.06 µg |  |
|  | M2 | Egr2 | APC Anti-Mouse Egr2 | eBioscience | 17-6691-80 | 0.35 µg |  |
| MDSCs | Pan MDSCs | Gr-1 | PE anti-mouse Gr-1/Ly6G | R&D | FAB1037P | 10 µL |  |
|  |  | CD11b | PE/Cy7 anti-mouse CD11b | Biolegend | 101215 | 0.25 µg |  |
|  | M-MDSCs | Ly6c | PE/Dazzle™ 594 anti-mouse Ly-6C | Biolegend | 128043 | 0.06 µg |  |
|  | G-MDSCs | Ly6g | APC anti-mouse Ly-6G | Biolegend | 127613 | 0.06 µg |  |
| Abbreviations: APC, Allophycocyanin; CD, Cluster of Differentiation; Cy, Cyanine; F4/80, EGF-Like Module-Containing Mucin-Like Hormone Receptor-Like 1; Egr2, Early Growth Response Protein 2; G-MDSCs, Granulocytic MDSCs; Gr-1, Myeloid Differentiation Antigen; Ly6c, Lymphocyte Antigen 6 Complex, Locus C1; Ly6G, Lymphocyte Antigen 6G; IFNγ, Interferon Gamma; MDSCs, Myeloid Derived Suppressor Cells; M-MDSCs, Monocytic MDSCs; NKT, Natural Killer T Cells; PE, Phycoerythrin; PerCP, Peridinin Chlorophyll Protein Complex; TCR, T Cell Receptor | | | | | | | |
